# Fertility awareness among Chinese university students: scale development and cross-sectional study

**DOI:** 10.3389/fpubh.2026.1818259

**Published:** 2026-05-20

**Authors:** Changmin Niu, Xueyu Lu, Yue Ren, Fan Yang, Shikun Ma, Yawen Yu, Yuxin Gao, Nuo Shen, Jinhua Shu

**Affiliations:** 1 Department of Nursing, Affilited Hospital of Yangzhou University, Yangzhou University, Yangzhou, Jiangsu, China; 2 School of Nursing, Faculty of Medicine, Yangzhou University, Yangzhou, Jiangsu, China; 3 School of Basic Medical Sciences & School of Public Health, Faculty of Medicine, Yangzhou University, Yangzhou, China; 4 Yangzhou hospital of Traditional Chinese Medicine, Yangzhou, Jiangsu, China

**Keywords:** fertility awareness, influence factors, level classification, reliability and validity, university students

## Abstract

**Background:**

Global fertility rates are declining. In China, the total fertility rate dropped sharply from 5.59 in 1971 to 1.15 in 2021. Chinese university students often lack adequate fertility awareness, yet no validated instrument exists to assess this construct in this population, despite its recognized importance for informed reproductive decision-making.

**Methods:**

The Fertility Awareness Scale for University Students (FASUS) was developed through literature search, semi-structured interviews, and two rounds of Delphi expert consultation. Psychometric properties were tested using exploratory and confirmatory factor analysis, Cronbach's α, split-half reliability, and test-retest reliability. A cross-sectional survey of 750 students examined fertility awareness levels and influencing factors via univariate analysis and multiple linear regression.

**Results:**

The final FASUS Scale consisted of 22 items across three dimensions: fertility-related knowledge, reproductive health attitudes, and reproductive health skills, with a cumulative variance contribution rate of 67.952%. The scale demonstrated strong reliability (Cronbach's α = 0.97, split-half reliability = 0.885, test-retest reliability = 0.981). A survey of 750 students revealed an average fertility awareness score of 78.01 ± 16.44, indicating a low to moderate level. Key influencing factors included gender, ethnicity, education level, only-child status, and prior fertility knowledge education.

**Conclusion:**

This study utilized a self-developed scale to assess fertility awareness among Chinese university students. The results showed that the fertility awareness level of Chinese university students was generally low. We suggest that health care institutions implement targeted intervention measures to improve reproductive health outcomes.

## 1 Introduction

Fertility constitutes a fundamental component of population dynamics, critically influencing both demographic quantity and quality (1). Recent data from The Lancet reveal a dramatic global decline in total fertility rates (TFR), decreasing from 4.84 in 1950 to 2.23 in 2021 (2). Projections indicate this downward trajectory will persist, with anticipated TFRs of 1.83 by 2050 and 1.59 by 2100, placing most nations below population replacement levels.

China, the world's second most populous nation (≈1.417 billion), has experienced particularly striking fertility reduction, with TFR plummeting from 5.59 in 1971 to 1.15 in 2021 (3). Despite progressive policy interventions including the 2016 universal two-child policy and 2021 three-child policy, fertility rates remain persistently low (4).

University students represent the primary future reproductive cohort. While studies confirm their general fertility intentions (5), significant knowledge gaps exist regarding fertility decline risks and infertility factors. This awareness deficit frequently leads to delayed childbearing decisions, missed optimal reproductive windows (25–30 years for women), and elevated infertility risks (6, 7). Many students prioritize education and career development without recognizing the accelerated fertility decline after age 35 (8). Furthermore, insufficient understanding of fertility determinants (e.g., age, lifestyle factors, sexually transmitted infections) often results in missed family planning opportunities and potential lifelong infertility (9–12).

The concept of fertility awareness (FA) was formally defined in the 2017 International Glossary on Infertility and Fertility Care as “understanding reproduction, fertility potential, and associated personal risk factors (e.g., advanced maternal age, sexual health factors, lifestyle choices) and non-personal risk factors (e.g., environmental exposures), while recognizing social and cultural influences on family planning decisions” (13). This standardized definition underscores FA's global significance for clinicians, researchers, and policymakers.

Valid FA assessment remains challenging. While Lampic's (11) Fertility Awareness Questionnaire represents the most widely used instrument, it exhibits notable limitations including female-centric focus and lack of quantitative criteria. Subsequent scales by Meissner (14) and Virtala (15) similarly fail to provide comprehensive FA assessment. Moreover, existing international tools primarily rely on unvalidated self-report measures. These limitations motivated our development of a culturally adapted Fertility Awareness Survey for University Students (FASUS) for the Chinese context.

Current Fertility Awareness research on Chinese university students remains scarce, with existing studies predominantly focusing on specialized populations (e.g., fertility treatment patients, gynecological cases) (16–18). To address this gap, we developed a psychometrically validated FASUS scale (See Supplementary Table 1 for details) and applied the FASUS scale along with general population questionnaire for investigation: (1) Assess current fertility awareness levels among Chinese university students, (2) Identify key factors associated with fertility awareness, and (3) Provide evidence-based targets for future FA improvement interventions.

## 2 Methods

All methods used in this study were performed in accordance with the ethical principles of the Declaration of Helsinki for medical research involving human subjects. This three-phase study received ethical approval from the School of Nursing at Yangzhou University (Approval No. YZUHL20220058, See Supplementary Figure 1 for details). All procedures adhered strictly to medical ethics standards, upholding principles of voluntary participation, impartiality, and confidentiality throughout the research process. All participants in the study had given written informed consent before participating in this research, and were aware that their participation was voluntary and that they could withdraw at any time without any consequences.

### 2.1 FASUS Scale development

The scale in this study was constructed based on the Information-Motivation-Behavioral Skills (IMB) model. Through systematic literature review, semi-structured interviews, Delphi expert consultation and item selection, a preliminary draft of the University Students' Fertility Awareness Scale was developed.

**Figure 1:**
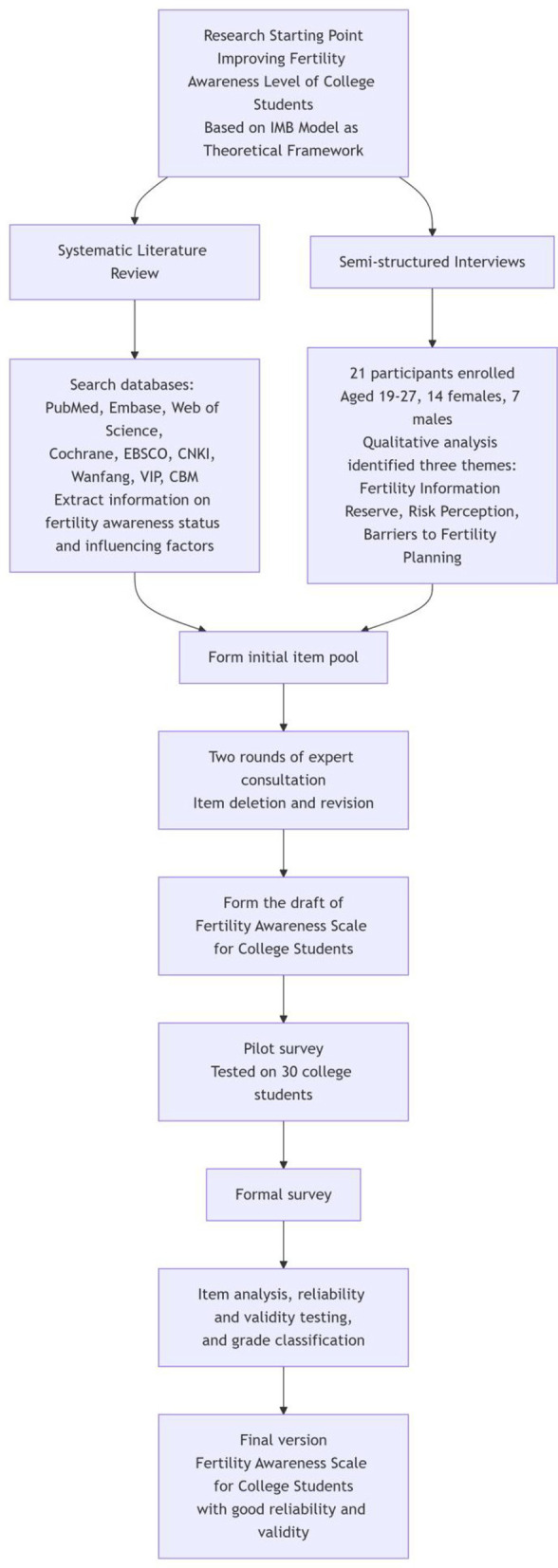
Flowchart of scale development process.

(1) Item pool development: Systematic searches were conducted in Chinese and English databases including PubMed and CNKI, yielding 21 articles from which fertility awareness items were extracted (19). Semi-structured interviews were also performed with 21 unmarried and childless university students (aged 19–27 years), and three themes (fertility knowledge, risk perception, and barriers to childbearing plans) were identified through thematic analysis. The interview outline is shown in Table 1. Based on the literature and interviews, an initial item pool with 3 first-level and 38 second-level indicators was formed (20). (See Supplementary Table 1 for details.).
(2) Delphi expert consultation: Seventeen experts in obstetrics, gynecology, and reproductive health participated in two rounds of Delphi consultation. Item importance was rated on a 5-point Likert scale, with retention criteria of mean ≥4 and coefficient of variation ≤ 0.3. Evaluation indicators included experts' positive coefficient, authority coefficient (Cr), and Kendall's W coefficient of concordance (21).
(3) Preliminary experiment: A preliminary investigation was conducted involving 30 university students to assess the clinical feasibility of the FASUS scale (first draft) and ensure the clarity and comprehensibility of its semantics. The detailed process table for constructing the scale is shown in Figure 1.

**Table 1:** Interview outline.

Serial number	Interview topics
(1)	Can you share your views on fertility?
(2)	Do you understand what fertility is?
(3)	Which age group do you think women have the highest/beginning to decline in fertility?
(4)	Do you plan to have children in the future?
(5)	Do you think how many children is the ideal number? What are your thoughts on the gender combination of your child?
(6)	What is your expected reproductive age?
(7)	What factors have influenced your views and choices about childbirth?
(8)	Based on your current student status, do you have any concerns or anxieties about childbirth?
(9)	Do you have any understanding of infertility?
(10)	Do you understand what factors affect fertility?
(11)	How did you learn about fertility knowledge?
(12)	If you or your partner are infertile, what would you do?

### 2.2 FASUS scale verification

The study recruited 420 participants from five universities in Jiangsu Province between May and July 2023 using convenience sampling method. Data collection was conducted through the Questionnaire Star platform following informed consent procedures. Inclusion criteria required participants to be: (1) Full-time university students, (2) Aged 18 years or older, and (3) Born in mainland China. Exclusion criteria comprised: (1) Married status or parenthood, and (2) Any language, auditory, or cognitive impairments that might affect survey comprehension. From the initial distribution of 450 questionnaires, 420 valid responses were obtained (response rate: 93.3%), constituting the final analytical sample.

The research group randomly divided the 420 participants into two groups. They used the received 210 samples (Sample 1) for item analysis and exploratory factor analysis. The 210 samples (Sample 2) were used for confirmatory factor analysis. All the samples were used for reliability analysis. Table 2 presents the complete demographic characteristics of the study participants.

**Table 2:** Demographics characteristics of participants (*n* = 420).

Project		Number of cases	Percentage
Gender	Male	140	33.30%
	Female	280	66.70%
Age	18	38	9%
	19	91	21.70%
	20	135	32.10%
	21	54	12.90%
	22	29	6.90%
	23	23	5.50%
	24	15	3.60%
	25	8	1.90%
	26	11	2.60%
	27	7	1.70%
	28	9	2.10%
Nation	Han nationality	408	97.10%
	Minority nationality	12	2.90%
Religious belief	NO	414	98.60%
	YES	6	1.40%
Education	Junior college	69	16.40%
	Undergraduate	288	68.60%
	Postgraduate	53	12.60%
	Doctoral students	10	2.40%
Target education	Undergraduate course	108	25.70%
	Postgraduate	236	56.20%
	Doctoral students	62	14.80%
Major	Science and Engineering	61	14.50%
	Medicine	239	56.90%
	Agronomy	57	13.60%
	Liberal arts	50	11.80%
	Art category	12	2.90%
Birthplace	Town, city	197	46.90%
	Rural area	223	53.10%
The only child	NO	218	51.90%
	YES	202	48.10%
Single-parent family	NO	389	92.60%
	YES	31	7.40%
Annual household income (¥)	< 50,000	71	16.90%
	50,000–100,000	149	35.50%
	100,000–300,000	167	39.85
	300,000–500,000	21	5%
	>500,000	12	2.90%
Sexual orientation	Uncertain	44	10.50%
	Bisexual	22	5.20%
	Homosexuality	13	3.10%
	Heterosexuality	341	81.20%
Relationship	Single	293	69.80%
	Being in love	127	30.20%

#### 2.2.1 Item analysis

The following steps were adopted for each method to refine the dataset based on the Item Analysis criteria. (1) Critical Ratio Method (CR Value): The CR value for each item was calculated by comparing the differences between the extreme groups using an independent samples *t*-test. Items with CR < 3 or *P* > 0.05 were deleted (21). (2) Correlation Coefficient Method: The corrected item-total correlation coefficient (r) was computed for each item. Items with r < 0.4 or *P* > 0.05 were deleted. (3) Cronbach's Alpha Method: The change in Cronbach's alpha (α) was observed after removing a specific item. Items whose Cronbach's α increases after deletion were removed. The application of these criteria systematically refined the dataset ensuring that it met item analysis standards.

#### 2.2.2 Reliability analysis

Cronbach's α was used to assess the internal consistency of FASUS (22); To assess split-half reliability, the scale entries were divided into two halves and scored accordingly based on the responses obtained from the subjects. Then, the correlation coefficient (r) was calculated between the scores obtained from the two halves of the questionnaire. A split-half reliability coefficient of at least 0.7 indicates a satisfactory level of internal consistency; Fifty respondents were randomly selected to undergo re-evaluation after 2 weeks, and the r was calculated based on the results of the two measurements. The criteria were based on retest reliability of ≥0.78 and *P* < 0.05 (23).

#### 2.2.3 Validity Analysis

To assess content validity, experts were invited to evaluate the relevance of each term in the FASUS scale to its respective dimension. The Content Validity Index (CVI) was calculated both at the item level (I-CVI) and the scale level (S-CVI), with I-CVI ≥0.70 and S-CVI ≥0.90 indicating acceptable content validity (24); Construct validity:

(1) Exploratory factor analysis (EFA): The varimax orthogonal rotation method was used for analysis following the principle of cumulative variance contribution rate >60% and eigenvalue >1. Factor loadings were required to be ≥0.50, with no double load entry (25).
(2) Confirmatory factor analysis (CFA): CFA was assayed using maximum likelihood estimation to validate the factor structure of the scale (26, 27). The following indices of model fitness were used: chi-squared to the degree of freedom ratio (CMIN/df), goodness of fit index (GFI), comparative fit index (CFI), root mean square error of approximation (RMSEA), and root mean square residual (RMR), with CMIN/df ≤ 3.0, RMR ≤ 0.05, RMSEA ≤ 0.08, GFI >0.90, or CFI ≥0.90, indicating a suitable test model (28).

FASUS scale grade division: SPSS 26.0 software was used for sample cluster analysis and discriminant analysis to grade the scale.

### 2.3 Investigation of current situation and influencing factors

#### 2.3.1 Survey tools

(1) General Information Questionnaire: The General Information Questionnaire including age, gender, educational level, profession, sexual orientation, relationship status, intention to have children, expected number of children to have, expected age for having the first/ last child, educational attainment, and sources of knowledge acquisition, etc;

(2) FASUS scale: The final FASUS scale developed in this study consists of 22 items across three dimensions: fertility-related knowledge, reproductive health attitudes, and reproductive health skill. Each item is scored using the five-point Likert scale, with options ranging from “completely in line” (5 points), “mostly in line” (4 points), “uncertain” (3 points), “mostly not in line” (2 points), to “completely not in line” (1 point). The higher the score on this scale, the higher the reproductive awareness level of university students. The detailed scale items can be found in Supplementary Table 1.

The questionnaire survey was conducted via the Questionnaire Star platform (https://www.wjx.cn). The platform generates the corresponding two-dimensional code when publishing the questionnaire. Participants were required to visit the “I agree to participate in this survey” page. The questionnaire page is only accessible to applicants who choose “yes”.

#### 2.3.2 Participants and sampling

This cross-sectional survey was conducted among mainland Chinese university students from July to December 2023. The original data were obtained from the Questionnaire Star system and collated. Invalid scales were excluded (samples with consistent or wavy answer options were not included) to guarantee data reliability and accuracy. Of the 810 questionnaires distributed, 60 were eliminated and 750 were successfully recovered with an effective recovery rate of 92.6%.

#### 2.3.3 Statistical methods

Statistical analysis was conducted using IBM SPSS version 26.0 and AMOS version 24.0. The differences in FA scores among Chinese university students were analyzed using a *t*-test and non-parametric test. Multiple linear regression was applied to evaluate factors influencing FA in Chinese university students. *P* < 0.05 was considered statistically significant.

## 3 Results

### 3.1 Formulate the initial version of the FASUS scale

A total of 21 studies were included in the literature review, showing that university students' fertility awareness was generally low to moderate, with gender and major as main influencing factors. Semi-structured interviews with 21 unmarried and childless university students (14 females, 7 males, aged 19–27 years) revealed three themes: insufficient fertility knowledge with limited access channels, inadequate perception of fertility risks (with females more concerned about age), and childbearing plans hindered by economic, academic, career, and partner-related factors. Based on the literature and interviews, an initial item pool consisting of 3 first-level and 38 second-level indicators was formed.

Seventeen experts completed two rounds of Delphi consultation (100% response rates both rounds; authority coefficients of 0.853 and 0.867; Kendall's W of 0.488 in the second round indicating good agreement). After the first round, 7 items were modified, 7 deleted, 5 added, and 2 merged; after the second round, expert consensus was reached. A preliminary scale with three dimensions (fertility-related knowledge, reproductive health attitude, reproductive health skills) and 30 items was finally developed.

### 3.2 Development and verification of the FASUS scale

#### 3.2.1 Item analysis

Using the 210 participants in Sample 1. Through rigorous psychometric evaluation employing three distinct item-reduction methods, we eliminated eight items (9, 12, 16, 20, 21, 25, 26, 28), yielding a final 22-item instrument. This refinement process aligns with established scale development practices to optimize measurement efficiency and validity.

#### 3.2.2 Exploratory factor analysis (EFA)

**Figure 2:**
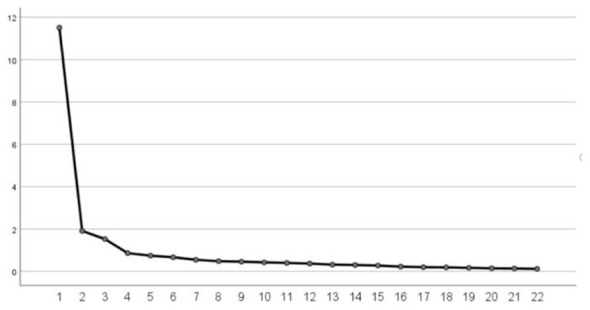
Scree plot.

EFA was performed on Sample 1 (*n* = 210). The Kaiser-Meyer-Olkin (KMO) measure was 0.930, and Bartlett's test of sphericity was significant (χ^2^ = 3,689.974, *P* < 0.001), indicating suitability for factor analysis. Principal component analysis with varimax rotation extracted three factors with eigenvalues >1, corresponding to the three hypothesized dimensions: fertility-related knowledge (11 items), reproductive health attitudes (7 items), and reproductive health skills (4 items). The cumulative variance explained was 67.952%. Factor loadings ranged from 0.601 to 0.842, with no cross-loadings. The scree plot is shown in Figure 2.

#### 3.2.3 Confirmatory factor analysis (CFA)

**Figure 3:**
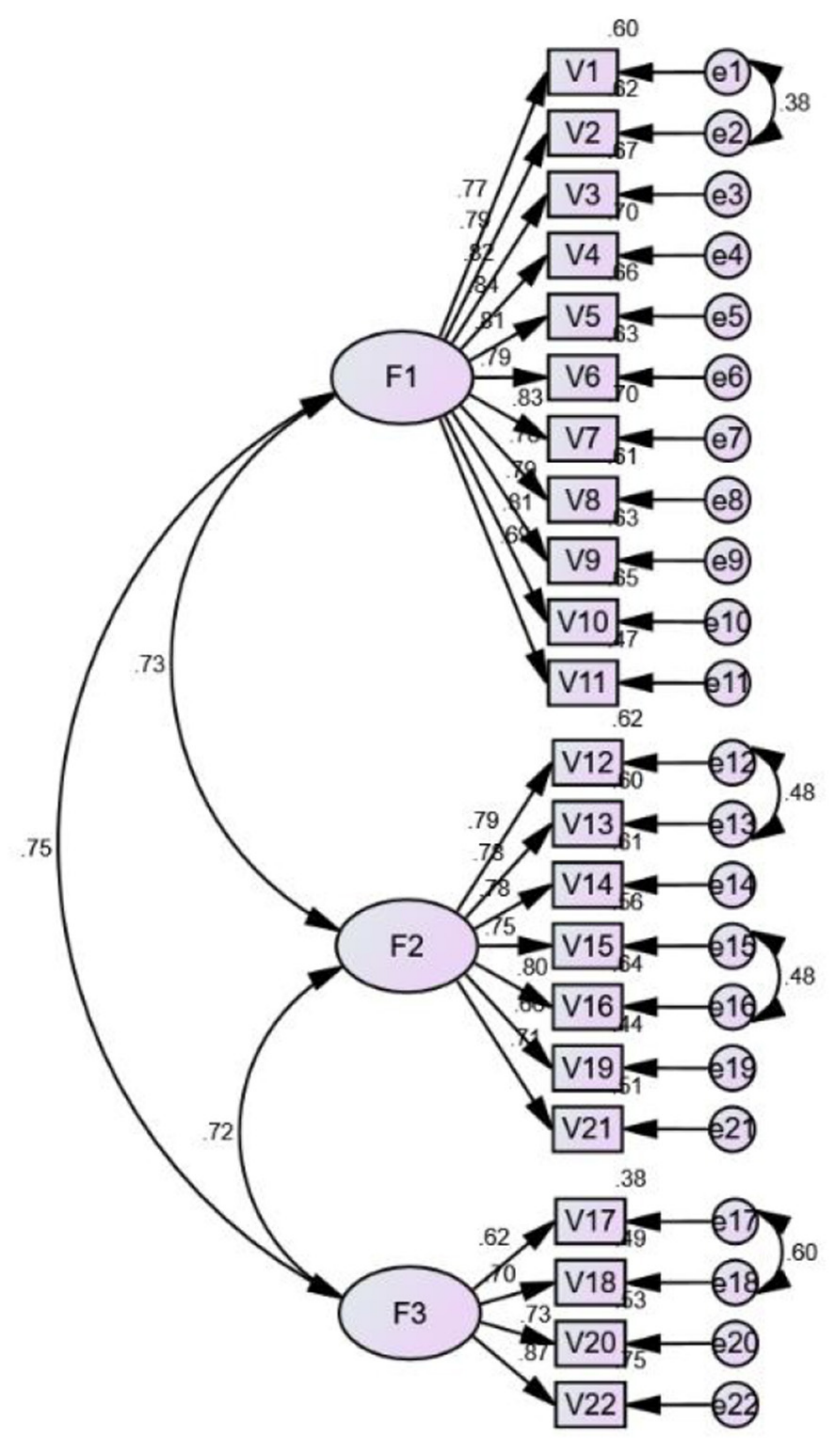
Modified model.

CFA was conducted on the independent Sample 2 (*n* = 210) to test the three-factor structure identified by EFA. The model yielded the following fit indices: CMIN/df = 2.624, CFI = 0.909, RMR = 0.046, RMSEA = 0.088, and GFI = 0.817. While the CFI and CMIN/df met conventional thresholds for acceptable fit (≥0.90 and ≤ 3.0, respectively), the RMSEA (recommended ≤ 0.08) and GFI (recommended ≥0.90) fell slightly below the ideal cutoffs. Overall, the three-factor structure is acceptable, the modified model is presented in Figure 3, and factor loadings are shown in Table 3.

**Table 3:** Factor loadings from exploratory factor analysis (rotated component matrix).

Item	Factor load		
	Factor 1	Factor 2	Factor 3
4. I understand that the best age for female fertility is around 25–30 years old	0.842		
7. I understand that women are most likely to conceive during ovulation	0.827		
8. I understand that women 's ovulation time during the menstrual cycle is about 14 days before the next menstruation (Normal menstrual cycle)	0.775		
3. I understand that healthy women have fertility in their lifetimes from the first ovulation to the last	0.768		
5. I understand that women 's fertility declines sharply after the age of 35	0.751		
6. I understand that age is an important factor affecting fertility	0.741		
10. I understand that repeated abortion in women can lead to infertility	0.690		
9. I understand that infertility can be divided into male factor infertility, female factor infertility and unexplained infertility	0.672		
1. I understand that fertility, also known as fertility and fertility, refers to the physical ability of both partners to give birth to live babies	0.664		
2. I understand fertility assessment mainly through the history, occupation, diet, living environment, women 's ovulation, fallopian tube function and ovarian function and male semen, such as several systematic assessments	0.661		
11. I understand that assisted reproductive technology (ART) is through a variety of interventions, procedures, surgery, and technology to achieve reproduction, to treat different forms of reproductive disorders and infertility	0.649		
13. I will protect my fertility by actively adjusting my lifestyle (balanced diet, smoking cessation, alcohol restriction, regular exercise, regular work and rest, etc.).		0.799	
12. I attach great importance to protecting my fertility		0.768	
16. I would like to receive education on reproductive health and fertility protection		0.750	
19. I adhere to a healthy diet		0.745	
14. I think it is very important to carry out professional fertility education on university campuses		0.716	
15. I hope to learn more about reproductive health through a variety of ways		0.696	
21. I will avoid exposing myself to environmental factors that can lead to low fertility or infertility		0.640	
18. I never smoke			0.861
17. I've never been an alcoholic			0.826
20. I will adjust my emotions through some activities to relieve the pressure in life and study			0.675
22. I take protective measures during sexual behavior to avoid sexually transmitted diseases			0.601

#### 3.2.4 Reliability and validity

(1) Content validity: The item-level content validity index (I-CVI) ranged from 0.820 to 1.000, and the scale-level CVI (S-CVI) was 0.964, indicating excellent content validity.

(2) Reliability: The Cronbach's α coefficient for the total scale was 0.970. The split-half reliability was 0.885, and the 2-week test-retest reliability (*n* = 50) was 0.981. These values indicate good internal consistency and temporal stability.

The final FASUS scale consists of 22 items across three dimensions.

### 3.3 Classification of scale scores

Cluster analysis was performed on the FA scores from 420 participants using K-means clustering, with dimension scores and total scale scores serving as classification variables. After 13 iterations, the optimal solution identified four distinct FA categories (*P* < 0.01). The classification yielded the following distribution: Grade I (22–54 points, *n* = 58), Grade II (55–70 points, *n* = 121), Grade III (71–85 points, *n* = 110), and Grade IV (86–110 points, *n* = 131). Discriminant function analysis demonstrated excellent classification accuracy (97.9%), confirming the validity of the score ranges for each grade (Table 4).

**Table 4:** Scale level classification (*n* = 420).

Level	Designation	Score interval (points)	Number of people (%)	Mean ±standard deviation
I	Extremely low level	22–54	56 (13.33)	46.47 ± 6.66
II	low level	55–70	120 (28.57)	63.16 ± 4.36
III	Moderate level	71–85	115 (27.38)	77.95 ± 4.46
IV	High level	86–110	129 (30.71)	93.27 ± 5.89

### 3.4 Analysis of fertility awareness level and influencing factors

#### 3.4.1 Demographic characteristics

A total of 750 students (306 males, 40.8%; 444 females, 59.2%) aged 18–32 years (mean 20.69 ± 2.23) participated. By education: undergraduate 410 (54.7%), junior college 245 (32.7%), master's 79 (10.5%), doctoral 16 (2.1%); 399 (53.2%) intended to pursue higher degrees. Medical majors 267 (35.6%), non-medical 483 (64.4%). Urban 351 (46.8%), rural 399 (53.2%). Only children 433 (57.7%), with siblings 317 (42.3%). Heterosexual 661 (88.1%). Relationship status: single 547 (73.2%), in a relationship 200 (26.8%).

#### 3.4.2 The fertility intentions and fertility knowledge sources of university students

When asked to rate the importance of having children on a scale of 0–10 (where 0 = “not crucial” and 10 = “extremely crucial”), the overall average score was 5.38. Concurrently, 398 students (53.1%) expressed a clear intention to have children. Regarding childbearing age, the vast majority of students (64.8%) expected to have their first child between 25 and 29 years of age, while 117 students (29.4%) expected to do so between 30 and 34 years of age (See Table 5 for details).

**Table 5:** The fertility intentions of university students.

Item	Classification	Number of cases (male/female)	Percentage
Intention to have children (*n* = 750)	Yes	398 (203/195)	53.1
	No	231 (65/166)	30.8
	Uncertainty	121 (38/83)	16.1
Expected age of the first child (*n* = 398)	≤ 24	18 (12/6)	4.5
	25–29	258 (139/119)	64.8
	30–34	117 (51/66)	29.4
	≥35	5 (1/4)	1.3

**Figure 4:**
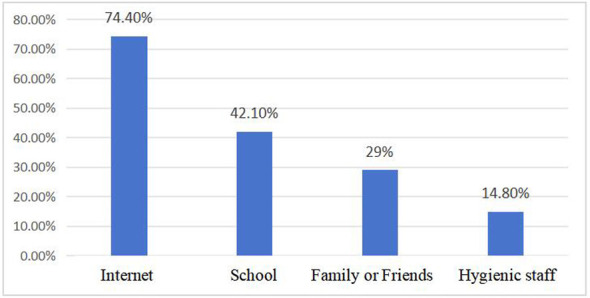
Ways for university students to obtain fertility knowledge.

The results regarding university students' access to fertility knowledge and their level of education showed that the majority of students (39.1%) reported having received only a little education related to fertility knowledge, 20% reported having received comprehensive education, and another 7.9% reported having received no such education at all. Regarding sources of fertility knowledge, the internet was the main channel for university students (74.4%), 42.1% obtained fertility knowledge from schools, 29% obtained it from family members or friends, and only 14.8% obtained fertility knowledge from healthcare professionals (See Table 6, Figure 4 for details).

**Table 6:** Fertility knowledge education level (*n* = 750).

Item	Classification	Number of cases	Percentage
Fertility knowledge education level	Have not received any education at all.	59	7.9
	I have received some education.	293	39.1
	I received a basic education.	248	33.1
	I have received a comprehensive education.	150	20

#### 3.4.3 Fertility awareness status in university students

**Figure 5:**
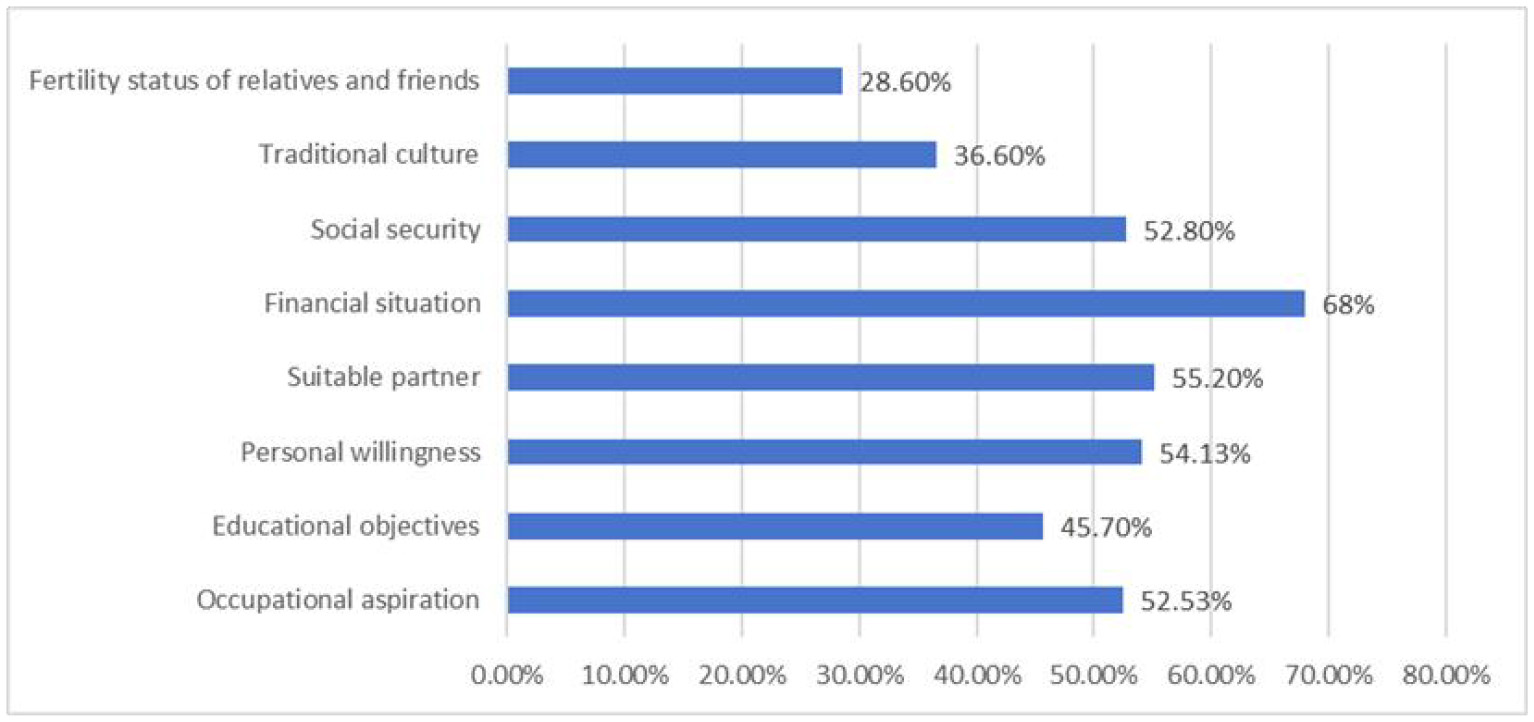
Factors affecting family planning and family development.

The average score of university students on the fertility awareness scale was (78.01 ± 16.44) points, with a score range of 22 to 110 points. There was a significant individual variation. The average item score was (3.54 ± 1.72) points. Among them, 68 cases (9.07%) were at the extremely low level I, 178 cases (23.73%) were at the low level II, 189 cases (25.2%) were at the medium level III, and 315 cases (42%) were at the high level IV. The average scores of the three dimensions of fertility-related knowledge, fertility health attitude, and fertility health skills were (37.68 ± 9.86) points, (24.61 ± 5.62) points, and (15.72 ± 3.73) points, respectively. In the open-ended question “What factors do you think affect your completion of the fertility plan and family building?”, the most frequently mentioned factor was economic conditions (68%), as detailed in Table 7 and Figure 5.

**Table 7:** The influence of general demographic data on fertility awareness (*n* = 750).

Item	Classification	FA score	Statistic	*P*
Gender	Male	75.18 ± 17.09	2.673^a^	0.001^***^
	Female	79.97 ± 15.70		
Nation	Han nationality	78.4 ± 16.29	0.682^a^	0.005^**^
	minority nationality	70.57 ± 17.75		
Religion	No	77.97 ± 16.492	1.346^a^	0.516
	Yes	81.56 ± 12.22		
Education	Junior college	72.73 ± 15.13	16.22^b^	< 0.001^***^
	Undergraduate	79.7 ± 16.61		
	Postgraduate	85.47 ± 15.18		
	Doctoral	78.94 ± 15.03		
Major	Medicine	85.78 ± 13.56	23.711^a^	< 0.001^***^
	Non-medical	73.72 ± 16.33		
Intention to improve academic qualifications	Yes	82.03 ± 15.09	8.348^a^	< 0.001^***^
	No	73.44 ± 16.74		
Origin	City	78.99 ± 17.07	2.659^a^	0.128
	Countryside	77.16 ± 15.84		
Only child	Yes	75.73 ± 17.53	23.707^a^	< 0.001^***^
	No	81.13 ± 14.28		
Single parent family	Yes	75.33 ± 15.47	1.698^a^	0.156
	No	78.28 ± 16.52		
Family annual income	< 50000	77.6 ± 15.05	1.24^b^	0.292
	50000-100000	79.4 ± 16.40		
	100000-300000	76.73 ± 16.56		
	300000-500000	80.43 ± 18.44		
	>500000	79.63 ± 17.65		
Sexual orientation	Heterosexuality	78 ± 16.63	0.572^b^	0.633
	Homosexuality	83 ± 15.48		
	Bisexuality	76.86 ± 14.74		
	Uncertain	77.2 ± 15.09		
Romantic relationship	Single	77.56 ± 16.34	1.434^a^	0.263
	In love	79.08 ± 16.76		

a is the F value, b is the *t* value, ^*^is significantly correlated at the 0.05 level (bilateral), ^**^ is significantly correlated at the 0.01 level (bilateral), and ^***^ is significantly correlated at the 0.001 level (bilateral). The scores of fertility awareness under different groups of related factors were analyzed by variance analysis, and the factors with statistical differences in the analysis of variance were compared by multiple comparisons afterward.

#### 3.4.4 Multivariate analysis of factors associated with fertility awareness

This study employed independent sample *t*-tests and one-way ANOVA to conduct a further analysis of the differences in FA among various factors in the general data, and conducted *post hoc* multiple comparisons for the factors with significant differences in homogeneity of variance. Ultimately, it was concluded that different genders, ethnicities, educational levels, majors, intentions to enhance educational attainment, whether being an only child, and the educational level of reproductive knowledge all had statistically significant differences in the scores of reproductive awareness among university students (See Table 7 for details).

Taking the FA score as the dependent variable, and including gender, ethnicity, educational level, major, whether there is an intention to upgrade educational level, being an only child, whether there is an intention to have children, and the educational level of knowledge about childbirth as independent variables, a multiple linear regression was conducted. The results showed that finally, five independent variables, namely gender, ethnicity, educational level, being an only child, and the educational level of knowledge about childbirth, entered the regression equation (F = 137.831, *P* < 0.05), with R^2^ = 0.576 and the adjusted R^2^ being 0.571. That is to say, these several variables can explain 57.1% of the variance of reproductive awareness (See Table 8 for details).

**Table 8:** The results of multiple linear regression analysis.

Item	B	SE	Beta	t	*P*
Gender	2.125	0.834	0.064	2.549	0.011
Nation	−4.071	1.848	−0.054	−2.202	0.028
Educational level	1.361	0.599	0.058	2.273	0.023
Only child	4.627	0.818	0.139	5.656	< 0.001
The educational level of fertility knowledge	1.696	0.49	0.091	3.462	0.001

## 4 Discussion

The scale developed in this study encompasses three dimensions: fertility-related knowledge, reproductive health attitudes, and reproductive health skills. Within the IMB model, fertility-related knowledge serves as a foundational prerequisite for effective behavior. This dimension consists of 11 items designed to assess university students‘ ability to acquire and utilize fertility and reproductive health information in daily life. A greater understanding of fertility-related issues increases the likelihood of individuals adopting health-seeking behaviors to improve reproductive outcomes (19). Reproductive health attitudes influence the extent to which university students prioritize reproductive health and engage in healthcare-seeking behaviors. Delayed childbearing intentions often stem from insufficient awareness of low fertility and infertility risks. Positive attitudes, as demonstrated in prior research (29), are generally associated with better health outcomes and greater adherence to health interventions. Regarding reproductive health skills, we incorporated both personal and lifestyle-related risk factors into our assessment. This allowed us to evaluate participants' awareness of modifiable risk factors (e.g., lifestyle choices) as well as non-modifiable influences on fertility. Compared to existing scales, the Fertility Awareness Scale for University Students (FASUS) is more closely aligned with theoretical frameworks and introduces novel perspectives in fertility awareness (FA) research (14, 15). It is worth noting that the confirmatory factor analysis yielded mixed fit indices. Although the CFI (0.909) and CMIN/df (2.624) indicated acceptable model fit, the RMSEA (0.088) and GFI (0.817) were slightly below ideal values. Future research should consider cross-validating the scale in larger and more diverse samples and exploring potential modifications to the item structure.

Cross-sectional studies have found significant differences in the level of Fertility awareness (FA) among individuals. Our data revealed that only 42% of the surveyed subjects achieved a high FA level, whereas 58% scored at a medium to low level, consistent with previous research findings (8). This outcome underscores a notable disparity in university students‘ reproductive knowledge, potentially impeding their ability to make scientifically informed reproductive decisions. Notably, the average score for fertility-related knowledge was the lowest at (3.42 ± 1.18) points. This domain covered aspects such as the definition, assessment, and risk factors of fertility, as well as the definition, risk factors, and treatment approaches for infertility. Such low scores may be closely associated with the traditionally conservative stance of Chinese education toward reproductive and sexual health topics (30). Conversely, the average score for reproductive health skills was the highest at (3.93 ± 1.20). This domain encapsulated a series of skills necessary for individuals to maintain fertility in daily life. The relatively high scores might be attributed to the emphasis placed by colleges and universities on students' daily behaviors and the cultivation of healthy habits. In contrast, the average score for reproductive health attitudes was comparatively low (3.51 ± 1.15). A positive health attitude is crucial as it facilitates the comprehension of health-related knowledge and the improvement of associated behaviors. Owing to the limited education on fertility—related matters, students remain largely unaware of the rising global and domestic incidence of infertility, which is increasingly affecting younger populations.

Generally, university students exhibit limited awareness of fertility-related issues. Fertility awareness among university students is influenced by factors such as gender, ethnicity, educational background, only-child status, and fertility knowledge education level. In real-world contexts, women tend to prioritize fertility, often demonstrating greater attention to and engagement in pre-pregnancy care (5, 31). Females are also more likely to pursue assisted reproductive technology or adoption in cases of infertility (32). Our results show that fertility awareness among university students is low to moderate, a finding that aligns with previous studies conducted in Poland, the United States, and South Korea (16, 19, 31). However, our study revealed several findings that differ from previous literature. First, unlike earlier studies that reported no significant ethnic differences (32), we found that Han students had significantly higher fertility awareness than ethnic minority students (*P* = 0.005), suggesting ethnicity may be an important factor in China's multi-ethnic context. Second, while previous research from Germany and Poland (19, 33), showed that medical students have higher fertility awareness than non-medical students, we observed no such difference (*P* > 0.05), possibly because medical students' prolonged training and awareness of family-planning conflicts offset their knowledge advantage (21). Third, a novel finding not previously reported is that non-only children scored higher than only children, a pattern likely shaped by China' s only-child policy. In contrast, the positive associations of educational attainment and prior fertility knowledge education with fertility awareness align with the IMB model (19) and are consistent with international evidence (32, 34). Female students scored higher than males, mirroring patterns in Sweden, Germany, and Argentina (5, 10, 33).

Fertility awareness is not merely an individual health concern but also deeply intertwined with national public health and sustainable population development. In China, despite a series of government policies aimed at encouraging higher birth rates, the fertility rate has remained persistently low. Some researchers have suggested that improving fertility literacy among future childbearing populations could be relevant in this context (35, 36). Whether tailored fertility education programs or curricula across different educational stages would effectively raise awareness levels remains to be tested. Future research, preferably using longitudinal or interventional designs with more representative samples, is needed to examine the potential impact of such educational efforts on fertility awareness, health behavior capabilities, and related outcomes.

## 5 Limitations and recommendations

(1) This study is a cross-sectional study, thus it is impossible to determine the causal relationship between the influencing factors (such as gender, educational level, whether being an only child, and previous reproductive education experiences) and fertility awareness (FA). Moreover, potential bidirectional or mediating paths (such as knowledge-acquisition-attitude) have not been tested. Future research should adopt a longitudinal cohort design or a randomized controlled method to evaluate the causal paths and the temporal evolution of fertility awareness. The use of mixed methods (such as qualitative research) can help understand the deep-seated influencing factors of fertility awareness;

(2) The development and validation of this research scale utilized a convenient sample from various universities in Jiangsu Province. However, it failed to adequately cover ethnic minorities as well as certain academic disciplines, majors, and geographical regions. This sampling method may introduce selection bias and limit its general applicability. Future research should adopt stratified, multi-stage probability sampling methods and conduct it in different provinces and types of institutions to facilitate comparisons among populations and the validation of the scale.

(3) The confirmatory factor analysis showed only adequate model fit, with RMSEA (0.088) and GFI (0.817) not reaching the conventional thresholds. Therefore, the three-factor structure of the FASUS should be interpreted with caution. Future studies should administer the scale to independent and more representative samples to further test its factorial validity and, if necessary, refine the item composition.

## 6 Conclusions

In this study, a cross-sectional survey using the University Students‘ Fertility Awareness Scale was conducted. Results indicated that the overall fertility awareness among university students remained at a low to moderate level. Factors associated with fertility awareness in this sample included gender, ethnicity, educational background, only-child status, and the level of prior fertility knowledge education. The FASUS demonstrated adequate psychometric properties for use in similar populations, though further validation with more diverse samples is warranted. Future studies with larger, more representative samples and longitudinal designs are needed to further examine the correlates of fertility awareness and to evaluate the effectiveness of educational interventions aimed at improving reproductive health knowledge and attitudes among university students. Medical and health institutions, colleges, and universities may consider assessing university students' fertility awareness and providing tailored guidance based on individual characteristics to support reproductive healthcare.

## Data Availability

The raw data supporting the conclusions of this article will be made available by the authors, without undue reservation.
