# Correction: Fertility awareness among Chinese university students: scale development and cross-sectional study

**DOI:** 10.3389/fpubh.2026.1920730

**Published:** 2026-07-10

**Authors:** Frontiers Production Office

**Affiliations:** Frontiers Media SA, Lausanne, Switzerland

**Keywords:** fertility awareness, influence factors, level classification, reliability and validity, university students

Authors Fan Yang and Jinhua Shu were erroneously assigned as equal contributing authors.

Authors Xueyu Lu and Yue Ren were erroneously omitted as equal contributing authors.

The original version of this article has been updated.

