# Supplementary material for: Fertility awareness among Chinese university students: scale development and cross-sectional study
Source: Front Public Health. 2026 May 20;14:1818259. doi: 10.3389/fpubh.2026.1818259 (PMC13230133; doi:10.3389/fpubh.2026.1818259)

Supplementary file

Table S1-Fertility Awareness Survey for University Students (FASUS)

| Item | Completely inconsistent | Inconsistent | Uncertain | Basically consistent | Completely consistent |
| --- | --- | --- | --- | --- | --- |
| 1. I understand that fertility, also known as fertility and fertility, refers to the physical ability of both partners to give birth to live babies. |  |  |  |  |  |
| 2. I understand fertility assessment mainly through the history, occupation, diet, living environment, women 's ovulation, fallopian tube function and ovarian function and male semen, such as several systematic assessments. |  |  |  |  |  |
| 3. I understand that healthy women have fertility in their lifetimes from the first ovulation to the last. |  |  |  |  |  |
| 4. I understand that the best age for female fertility is around 25-30 years old. |  |  |  |  |  |
| 5. I understand that women 's fertility declines sharply after the age of 35. |  |  |  |  |  |
| 6. I understand that age is an important factor affecting fertility. |  |  |  |  |  |
| 7. I understand that women are most likely to conceive during ovulation. |  |  |  |  |  |
| 8. I understand that women 's ovulation time during the menstrual cycle is about 14 days before the next menstruation (Normal menstrual cycle ). |  |  |  |  |  |
| 9. I understand that infertility can be divided into male factor infertility, female factor infertility and unexplained infertility. |  |  |  |  |  |
| 10. I understand that repeated abortion in women can lead to infertility. |  |  |  |  |  |
| 11. I understand that assisted reproductive technology (ART) is through a variety of interventions, procedures, surgery, and technology to achieve reproduction, to treat different forms of reproductive disorders and infertility. |  |  |  |  |  |
| 12. I attach great importance to protecting my fertility. |  |  |  |  |  |
| 13. I will protect my fertility by actively adjusting my lifestyle (balanced diet, smoking cessation, alcohol restriction, regular exercise, regular work and rest, etc). |  |  |  |  |  |
| 14. I think it is very important to carry out professional fertility education on university campuses. |  |  |  |  |  |
| 15. I hope to learn more about reproductive health through a variety of ways. |  |  |  |  |  |
| 16. I would like to receive education on reproductive health and fertility protection. |  |  |  |  |  |
| I've never been an alcoholic. |  |  |  |  |  |
| 18. I never smoke. |  |  |  |  |  |
| 19. I adhere to a healthy diet. |  |  |  |  |  |
| 20. I will adjust my emotions through some activities to relieve the pressure in life and study. |  |  |  |  |  |
| 21. I will avoid exposing myself to environmental factors that can lead to low fertility or infertility. |  |  |  |  |  |
| 22. I take protective measures during sexual behavior to avoid sexually transmitted diseases. |  |  |  |  |  |

**Figure S1-Ethical Approval Report**


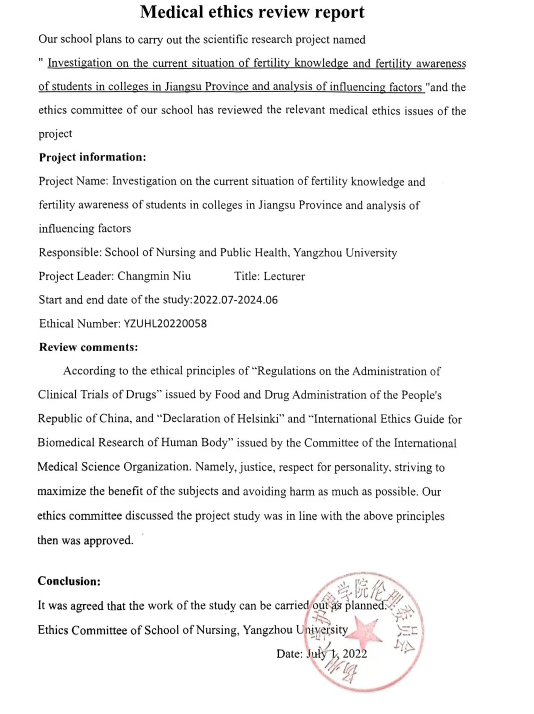

Supplement: Supplementary file 1 [file Table_1.docx]
